# Foam Structure Preservation during Microwave-Assisted Vacuum Drying: Significance of Interfacial and Dielectric Properties of the Bulk Phase of Foams from Polysorbate 80–Maltodextrin Dispersions

**DOI:** 10.3390/foods10061163

**Published:** 2021-05-22

**Authors:** Peter Kubbutat, Ulrich Kulozik, Jannika Dombrowski

**Affiliations:** 1 Chair of Food and Bioprocess Engineering, TUM School of Life Science, Technical University of Munich, Weihenstephaner Berg 1, 85354 Freising, Germany; Ulrich.Kulozik@tum.de (U.K.); Jannika.Dombrowski@rd.nestle.com (J.D.); 2 Nestlé Research, Société des Produits Nestlé SA, Route du Jorat 57, 1000 Lausanne, Switzerland

**Keywords:** vacuum drying, surfactant, polysaccharide, foam decay, resonant frequency

## Abstract

This study aimed at examining the cause of differences in the structure preservation of polysorbate 80–maltodextrin foams during microwave-assisted vacuum drying (MWVD) versus conventional vacuum drying (CVD). Aqueous dispersions of 3% polysorbate 80 and 0–40% maltodextrin were characterized for their dielectric and interfacial properties, and results were related to their drying performance in a foamed state. Surface tension and surface dilatational properties as well as dielectric properties clearly responded to the variation in the maltodextrin content. Likewise, the foam structure preservation during CVD was linked to the maltodextrin concentration. Regarding MWVD, however, foams collapsed at all conditions tested. Nevertheless, if the structure during MWVD remained stable, the drying time was significantly reduced. Eventually, this finding could be linked to the dielectric properties of polysorbate 80 rather than its adsorption kinetics and surface film viscoelasticity as its resonant frequency fell within the working frequency of the microwave drying plant.

## 1. Introduction

For the preservation of heat-sensitive biological material (e.g., bacterial cultures [1,2] enzymes [3] or pharmaceutical ingredients [4]), vacuum drying has evolved into a promising alternative to time- and energy-intensive freeze drying. Besides advantages in the specific energy demand [5] and drying time [6], vacuum drying was shown to yield products of increased storage stability as compared to freeze drying. The increased storage stability was linked to product shrinkage during dehydration [7], which, however, entails a prolongation of the third drying stage. In addition, due to the compactness of the dried product structure, its grindability is impaired. In this context, Ambros et al. [8] reported that microwave assistance can reduce the vacuum drying time by about 95%. This significant effect was related to the volumetric energy input of the microwaves allowing for a high mass transfer over all drying stages. Furthermore, controlled product aeration (i.e., foaming) prior to microwave-assisted vacuum drying (MWVD) was proposed to tackle the drawback of conventional vacuum drying (CVD) in terms of poor product grindability [9]. Additionally, the drying process can be accelerated by means of foaming. This can be attributed to the higher specific surface of an aerated versus a non-aerated product as well as the presence of lamellae, which act as capillaries during the drying process, transporting the water to the product’s surface [10].

Overall, MWVD of foamed materials was shown to display a promising concept. However, it is important to note that product formulation (e.g., foaming agent, protectant, carrier) and characteristics (e.g., foam characteristics) as well as process conditions (e.g., pressure level, microwave power level, drying protocol) need to be well adapted and controlled [11]. Therewith, foam structure stability and thus product quality, besides process efficiency, are ensured. In order to avoid detrimental foam collapse during drying and further optimize MWVD in terms of the drying time and resulting product quality, a more in-depth understanding of the interdependency of the above-mentioned influencing factors is required [12]. It was assumed that interfacial and/or dielectric properties of the material to be dried would play a key role. Thereby, the former allow for conclusions regarding adsorption kinetics and the stability of the interfacial film against mechanical stress (e.g., stretching) and are associated with foamability and foam stability [13,14]. The latter provide insights into the interaction of the material to be dried with the electromagnetic field of the microwave and are key for process understanding [15]. To the authors’ best knowledge, neither interfacial characteristics nor dielectric properties of surfactant–polysaccharide or protein–polysaccharide dispersions have been investigated in the context of microwave-assisted vacuum drying, thus far. Therefore, the objective of the present study was to evaluate these characteristics (i.e., surface tension and surface dilatational rheology, dielectric constant and loss factor, resonant frequency and quality factor) for polysorbate 80–maltodextrin dispersions and to correlate them with the samples’ drying performance in terms of foam structure preservation during MWVD versus CVD. In this model system, the non-ionic surfactant polysorbate 80 served as a foaming agent, whereas the polysaccharide maltodextrin was used to enhance foam stability by means of increasing the bulk viscosity. In addition, maltodextrin is widely applied as a protectant or carrier for sensitive biological material during drying [8,16,17]. The behavior of whey protein–maltodextrin-based matrices was examined in a further study [18].

## 2. Materials and Methods

### 2.1. Materials and Preparation of Sample Dispersions

Sample dispersions of 200 g each were prepared by blending 3.0% *w/w* polysorbate 80 (Tween 80; Gerbu Biotechnik GmbH, Heidelberg, Germany) with different amounts (i.e., 0.0–40.0% *w/w*) of maltodextrin DE-6 (MD; Nutricia GmbH, Erlangen, Germany) and dissolving the dry mix in deionized water (Milli-Q Integral 3, Merck KGaG, Darmstadt, Germany). To ensure full hydration, the sample dispersions were gently stirred using a magnet stirrer (Maxi Direct, Fisher Scientific GmbH, Schwerte, Germany) at 200 rpm for 12 h at 4 °C. Prior to the experiments, the sample dispersions were tempered at 20 °C with a water bath (F3, Fisher Scientific GmbH, Schwerte, Germany). 

Unless mentioned otherwise, measurements were performed at 20 °C and at least in triplicate using independent batches of sample dispersions. Error bars represent the calculated standard deviation.

### 2.2. Surface Tension and Surface Dilatational Properties of the Bulk Phase

Time-dependent evolution of surface tension σ was determined by the pendant drop method using the DSA100R (Krüss GmbH, Hamburg, Germany). A drop of 12 µL was formed at the tip of a capillary (d_i_ = 1.81 mm, Krüss GmbH, Hamburg, Germany) and its contour was monitored for 120 min. Extraction of surface tension was conducted by drop shape analysis using the software Advance (Krüss GmbH, Hamburg, Germany). 

Upon reaching quasi-equilibrium surface tension, surface dilatational properties were determined by the oscillating drop method. For this, the pendant drop at a drop age of 60 min was exposed to a sinusoidal oscillation with a frequency of 0.1 Hz for a duration of 100 s. The amplitude was set to 200–800‰ in order to achieve a change in surface area by 2.5–3.5%. Data fitting according to Lucasson and Van den Tempel [19] allowed for the estimation of surface dilatational elasticity E’ and surface dilatational viscosity E’’:

(1) $$E=\frac{\Delta \sigma }{\Delta A/A_{0}}$$

(2) $$E^{\prime }=\left|E\right|\cos\varphi$$

(3) $$E^{\prime \prime }=\left|E\right|\sin\varphi$$

where *E* is the complex viscoelastic modulus, $\Delta A/A_{0}$ is the amplitude of the surface area oscillation and *φ* is the phase angle between surface tension oscillation and surface area oscillation. The tangent *φ* was calculated by dividing *E’’/E’* according to Conde et al. [20].

### 2.3. Dielectric Properties of the Bulk Phase

Dielectric properties of the sample dispersions were measured with the µWaveAnalyser (Püschner GmbH & Co. KG, Schwanewede, Germany) over a broad temperature range from −40 to +40 °C. For this, 300 µL of sample dispersion was filled into a 1 mL glass vial, which was then sealed with a lock (1MLFBG, VWR International GmbH, Darmstadt, Germany). For each measurement point, the filled vial and the µWaveAnalyser were tempered at the respective target temperature within a climate chamber. After an equilibration time of 6 h, measurements were performed at a frequency between 2400 and 2500 MHz against an empty vial. Based on the response of the sample dispersion towards the emitted frequency, the software µWaveAnalyser Version 3.2.0 (Püschner GmbH & Co. KG, Schwanewede, Germany) calculates the dielectric constant ε’, the dielectric loss ε’’, the resonant frequency f_res_ and the quality factor Q [21]. The dielectric constant is a measure of the polarizability of a material, whereas the dielectric loss is a parameter for the physical conversion of electromagnetic radiation into heat. In addition, the loss factor tanδ describes the ability of the sample dispersion to take up energy from the electromagnetic field and is defined by the ratio of loss factor and dielectric constant [22]:

(4) $$\tan\delta =\frac{\varepsilon^{\prime \prime }}{\varepsilon^{\prime }}$$

In addition, the penetration depth PD can be calculated according to Equation (5) [23]:

(5) $$PD=\frac{\lambda_{2450MHz}}{2\cdot \pi }\frac{\sqrt{\varepsilon^{\prime }}}{\varepsilon^{\prime \prime }}$$

The penetration depth describes the depth until the power density has decreased to $\frac{1}{e}$ of its initial value and depends on the wavelength *λ*. In this study, the wavelength was assumed with 2450 MHz.

### 2.4. Foam Preparation

Foams were prepared by means of whipping with a commercial planetary mixer (KitchenAid ARTISAN 5KSM105PS, Whirlpool Corp., Greenville, United States of America) equipped with a wire whisk geometry (K45WW, Whirlpool Corp., Greenville, United States of America). Per foam, 150 g of sample dispersion was whipped for 15 min at 220 rpm. Immediately after foam formation, 15 g of foamed sample was gently transferred to a cylindrical crystallization glass with a diameter of 200 nm (VWR International GmbH, Darmstadt, Germany) and dried as described in Section 2.5.

The resulting foam properties and viscosity values of the investigated sample dispersions are published in a previous study [24].

### 2.5. Foam Drying and Product Characterization

Foams were dried by means of two different techniques: conventional vacuum drying (CVD) and microwave-assisted vacuum drying (MWVD). 

For CVD, a pilot freeze dryer (Delta 1-24LSC; Martin Christ Gefriertrocknungsanlagen GmbH, Osterode am Harz, Germany) was operated at a chamber pressure of 15 mbar and a shelf temperature of 20 °C. The foams were dried for 16 h. In case of foam collapse, drying was stopped 10 min after the collapse occurred. 

**Figure 1:**
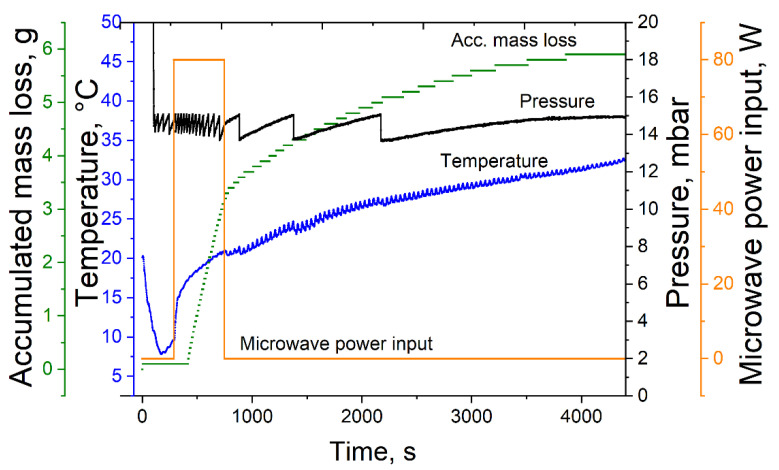
Exemplary microwave-assisted foam vacuum drying process at 15 mbar, 80 W microwave power input and a sample temperature of 20 °C.

For MWVD, the microwave drying plant µVac0150fd (Püschner GmbH & Co. KG, Schwanewede, Germany) was used. Process control was conducted with the software µWaveCAT (Püschner GmbH & Co. KG, Schwanewede, Germany). A typical MWVD process is shown in Figure 1. Similar to CVD, MWVD was performed at 15 mbar. The maximum temperature was limited to 20 °C, and a microwave power input of 80 W was set. The drying process was stopped 10 min after foam collapse or when no mass loss was detected during 10 consecutive minutes. For successfully dried samples, the drying time was between 45 and 90 min. The appearance of the resulting product structures was optically described.

## 3. Results and Discussion

### 3.1. Foam Drying

For the investigation of the interrelation between foam structure and drying behavior as a function of the heating technique, foams were prepared from polysorbate 80 at 3.0% *w/w* in combination with 10–40% *w/w* maltodextrin (MD). Thereby, an increase in the MD concentration led to an increase in the sample dispersion viscosity, which in turn resulted in a decrease in the overrun as well as the bubble size of the formed foams (results not shown). In terms of heating method, the foams were either subjected to conventional vacuum drying (CVD) or microwave-assisted vacuum drying (MWVD). For both drying processes, a maximum product temperature of 20 °C was set. Overall, this approach gave the opportunity to distinguish heating method-specific effects and to identify relevant product properties for a successful drying process.

**Figure 2:**
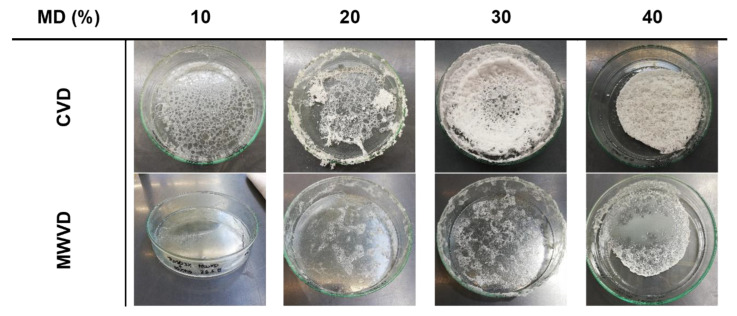
Impact of maltodextrin (MD) concentration and drying method on the appearance of the resulting product structures.

Figure 2 shows the different product structures obtained after either CVD or MWVD as a function of the MD concentration. Overall, both drying techniques, as well as MD content, had a clear impact on the appearance of the resulting product structures. For both drying techniques, foam structure preservation increased with increasing MD concentration, though CVD was clearly superior to MWVD. In the latter case, all samples collapsed during the drying process, yielding highly viscous liquids at the bottom of the sample containers. By contrast, by CVD, a foam-like structure was observed for samples containing 30 or 40% MD, whereas for 10 and 20% MD, foam collapse occurred during the first 30 min of drying. A better foam preservation during vacuum drying as a result of saccharide addition was also observed by Jangle and Pisal [25] investigating the impact of sucrose and mannitol on the vacuum foam drying of bovine serum albumin. The necessary amount of saccharide to obtain a foamy structure after the drying was comparable (>20% *w/v*). This is interesting because the viscosity of a 20% sucrose dispersion is expected to be lower than a 20% maltodextrin dispersion, and the viscosity should preserve the foam structure [9]. A reason for this difference might be that in the recent study, foam formation was conducted before the drying process was started. Thereby, the foam was destabilized while the pressure was decreased, whereas, in the study of Jangle and Pisal, the vacuum step was necessary to form the foam. Hence, in our study, the foam expanded even before it got heated, which resulted in higher mechanical stress.

Overall, the results show that CVD was better suited to preserve foam structures from polysorbate 80 in combination with MD than MWVD. This raised the question of the specific relationship between the type of energy input and the surface properties of polysorbate 80 with MD. In this context, this study aimed at distinguishing properties being relevant for structure preservation and successful drying. In order to close this knowledge gap, surface tension and surface dilatational properties, as well as dielectric properties of the sample dispersions, were determined. 

### 3.2. Surface Tension and Surface Dilatational Properties of the Bulk Phase

**Figure 3:**
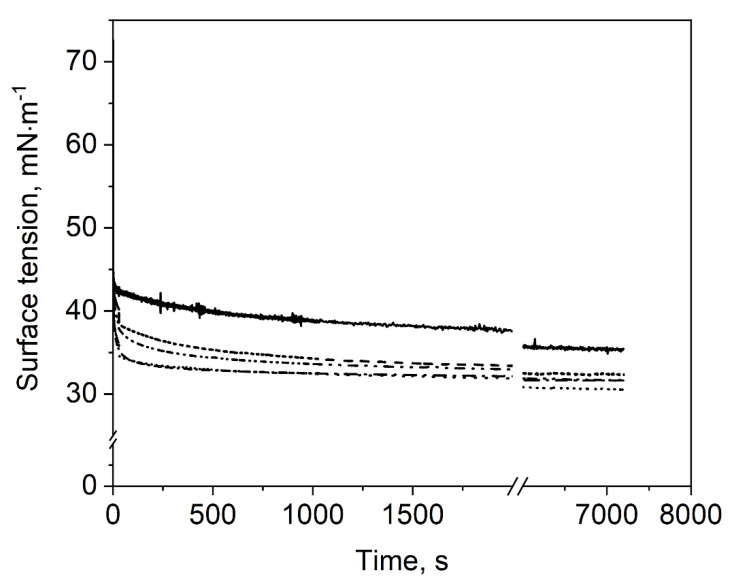
Impact of maltodextrin concentration on the time-dependent surface tension of polysorbate 80: (-) 0% MD, (- -) 10% MD, (∙∙∙∙) 20% MD, (-∙-) 30% MD, (-∙∙-) 40% MD.

Firstly, the evolution of surface tension of the different sample dispersions (i.e., polysorbate 80 with 0–40% MD) was measured for a period of 7200 s. The obtained results are displayed in Figure 3. The surface activity refers to the rate of initial surface tension decrease [26], which was calculated from the slope of the surface tension within the first 5 s. In general, all sample dispersions exhibited a relatively high surface activity, which is characterized by the fact that quasi-equilibrium surface tension was almost reached within the first few seconds. The slight further decrease in surface tension over time was considered rather insignificant as compared to the very initial decrease. 

It was observed that samples with MD showed higher surface activity and lower surface tension than the reference sample dispersion without MD. The surface activity increased from −6.0 at 0% MD to −7.4 mN·m^−1^·s^−1^ with 30% MD. The 40% MD samples showed a slightly lower value of −6.8 mN·m^−1^·s^−1^. Quasi-equilibrium surface tension of samples containing MD was around 33 mN·m^−1^, whereas for 0% MD, a value of 37 mN·m^−1^ was obtained. A possible explanation for this behavior could stem from the presence of surface-active molecules such as native starch lipids and proteins, originating from the maltodextrin manufacturing process [27,28]. However, the surface tension of MD dispersions with an MD content between 10 and 40% and without polysorbate 80 was reported to be in equilibrium between 71.2 and 72.5 mN·m^−1^ [24]. Hence, the contamination of MD with surface-active components seems not to be the reason for the observed differences in surface tension. Therefore, we assume that the high viscosity of 40% MD samples inhibited the polysorbate to move to the new surface and was therefore the reason for the slightly lower surface activity. Further, strong hydrogen bonds and hydrophobic interactions between polysorbate 80 and MD may change the surface activity of polysorbate 80 as also suggested by Semenova et al. [29]. Thereby, the surfactant might be modified *via* non-covalent interaction by extending the ethylene oxide group of polysorbate. Consequently, polysorbate 80–MD mixtures could lower the surface tension more than samples which contain only polysorbate 80. On the other hand, this might also be the reason for the slower adsorption of samples with 40% MD, as the interactions might result in a steric repulsion at the surface in the presence of high carbohydrate concentrations.

The lowest surface tension was obtained for sample dispersions containing 10 or 20% MD, whereas the surface tension was slightly higher for sample dispersions with 30 and 40% MD. This could be due to the increasing viscosity of the investigated sample dispersions with increasing MD concentration. Thereby, diffusion and adsorption of the surface-active molecules at the air/water interface might be hindered, resulting in a higher surface tension within the time frame investigated. Nevertheless, MD had an enhancing effect on surface activity and surface tension as compared to the reference sample dispersion without MD. 

Overall, the results show that surface activity and surface tension are not indicative for structure preservation during drying (Figure 2).

**Figure 4:**
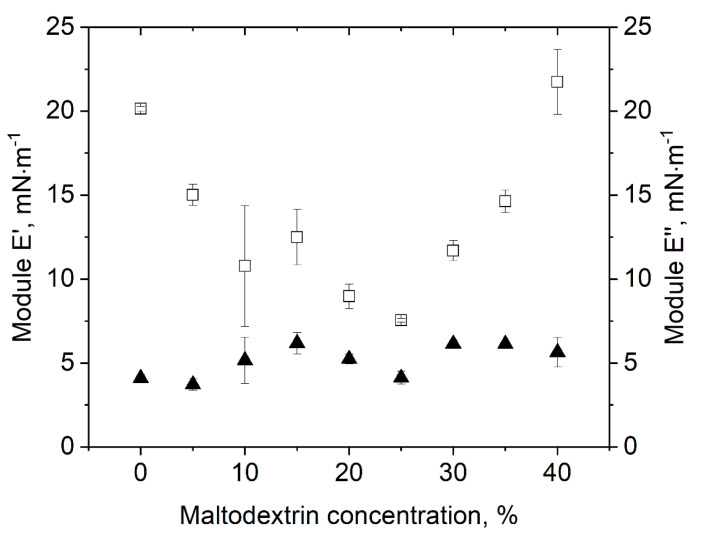
Impact of MD concentration on surface dilatational properties of polysorbate 80: (□) surface dilatational elasticity *E’*; (▲) surface dilatational viscosity *E’’* at an amplitude of 500‰.

Besides surface tension, the different sample dispersions were also characterized in terms of their surface dilatational properties. Surface dilatational properties are deemed indicative of surface film stability, which was assumed to be of importance in view of gas expansion under vacuum as well as bubble deformation during drying. The results on surface dilatational elasticity *E’* and surface dilatational viscosity *E’’* are shown in Figure 4 as a function of the MD concentration. In terms of *E’’*, no clear dependence on the MD concentration could be observed, though values slightly increased with increasing MD concentration from about 3.5 to 5.8 mN·m^−1^. Nonetheless, the very slight increase in *E’’* could be due to the increased total solids content of the sample dispersions with increasing MD concentration, which in turn showed a positive effect during drying. Unlike surface tension, surface dilatational properties and especially *E’* strongly depended on the MD content. By comparison, results on *E’* followed a “u” shape with a minimum at 25% MD. Thereby, the relative change in surface dilatational elasticity as a function of the MD concentration was quite high, with absolute values starting at 20.1 mN·m^−1^ at 0% MD, decreasing to 7.5 mN·m^−1^ at 25% MD and increasing again towards 21.7 mN·m^−1^ at 40% MD. This can also be described by tan(*φ*), where 0 represents a perfect elastic behavior of the film (Table 1) [20,30]. The surface showed most elastic behavior at low (tan(*φ*) = 0.2–0.24 at 0–5% MD) and high MD (tan(*φ*) = 0.28 at 40% MD), while for 20% MD, the surface exhibited the lowest elasticity (tan(*φ*) = 0.53). However, in comparison with protein-stabilized systems, the obtained tan(*φ*) for our system is quite high. For example, Beaza et al. [30] investigated the influence of different additives to β-lactoglobulin and obtained tan(*φ*) values of < 0.2. An explanation for this difference might be protein network formation by means of intermolecular interactions between protein molecules such as electrostatic interactions or hydrogen bonds. By contrast, small non-ionic surfactants such as polysorbate 80 are mainly interacting *via* hydrogen bonds. 

Overall, the surface of polysorbate-stabilized foams showed low elastic behavior, which could be problematic regarding the high mechanical stress during the drying process.

However, no clear correlation could be resolved between drying performance in terms of structure preservation and surface dilatational elasticity. 

The effect of better stability at higher total solid content could be explained as such that the high viscosity of the bulk phase prevents the bubbles to expand too quickly. During drying, the lamellae become very thin, and the surface area increases. This in turn might result in the occurrence of holes in the surface films whereby foam collapse is triggered, eventually. The higher MD content could slow this down and enable the foaming agent to cover the surface quickly enough to prevent foam collapse. In terms of conventional vacuum drying, an MD concentration of ≥ 30% seemed favorable in terms of formation of elastic surface films, resulting in structure preservation during drying. 

However, the results do not explain the observed differences between CVD and MWVD processes. Therefore, it was considered necessary to investigate in more depth the microwave-specific properties of the different sample dispersions. Therewith, it was aimed to better understand the underlying mechanisms allowing for specific conclusions regarding future product design.

### 3.3. Dielectric Properties of the Bulk Phase

**Figure 5:**
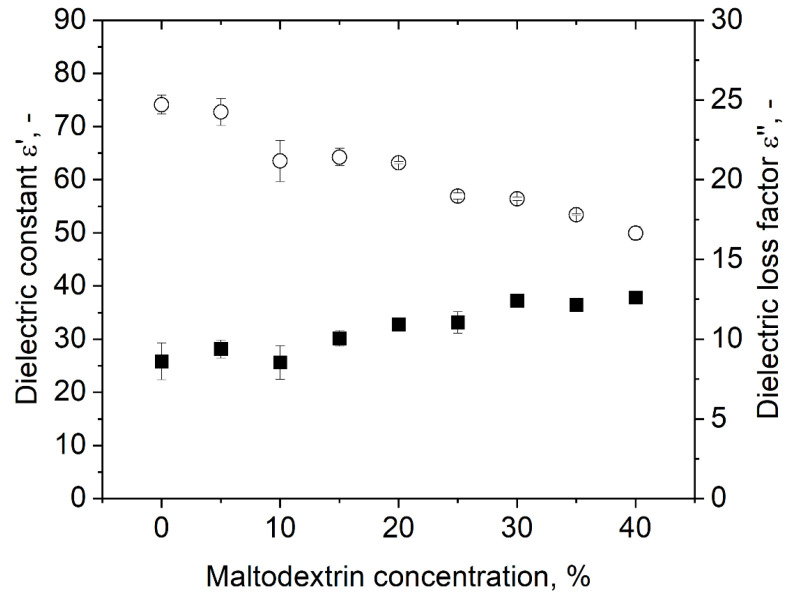
Impact of MD concentration on the dielectric properties of dispersions with 3% polysorbate 80. Symbols: (○) dielectric constant ε’, (■) dielectric loss ε’’.

The dielectric properties of polysorbate 80 dispersions with various contents of MD are shown in Figure 5. The dielectric constant decreased with increasing sugar concentration, while the dielectric loss factor slightly increased. A comparable trend for increasing carbohydrate concentration was also observed by Roebuck et al. [31] during their investigation of dielectric properties of carbohydrates using a microwave frequency of 3 GHz. They stated that the reason for the decrease in the dielectric constant was due to fewer polarizable dipole moments due to less free water in the samples. The increase in the dielectric loss factor with increasing carbohydrate content can be explained by a better stabilization of hydrogen bonds in the presence of carbohydrates. Haggis et al. [32] stated that due to better stabilized hydrogen bonds in the presence of organic molecules, the relaxation frequency of water is lowered, whereby the dielectric loss increases. Therefore, our findings are in accordance with our expectations and findings from the literature.

As a result of the increasing dielectric loss and decreasing dielectric constant, the value for tan (*δ*), which is a parameter for the ability of the material to converse radiation power into heat, increased (Table 2). Therefore, the samples were heated more efficiently with the higher sugar content. However, this might be problematic for the uniformity of the heating process: the more efficient the heating, the lower the penetration depth according to Equation (5). For the investigated samples, the penetration depth of the bulk decreased from 7.67 at 0% MD to 3.84 cm at 40% MD. Therefore, the characteristics of the heating are less volumetric with increasing MD because more energy is converted into heat within a shorter distance. As a result, the probability of hot and cold spot formation is increased. The bubbles are assumed to not be heated extensively because the bulk phase converts the energy much more efficiently than the air inside the bubbles. Consequently, the penetration depth into the foam will increase with higher overrun values, which means that the differences in the heating pattern are expected to be even higher. However, no differences in hot spot formation were detected, which was contributed to the foam expansion during the drying process.

**Figure 6:**
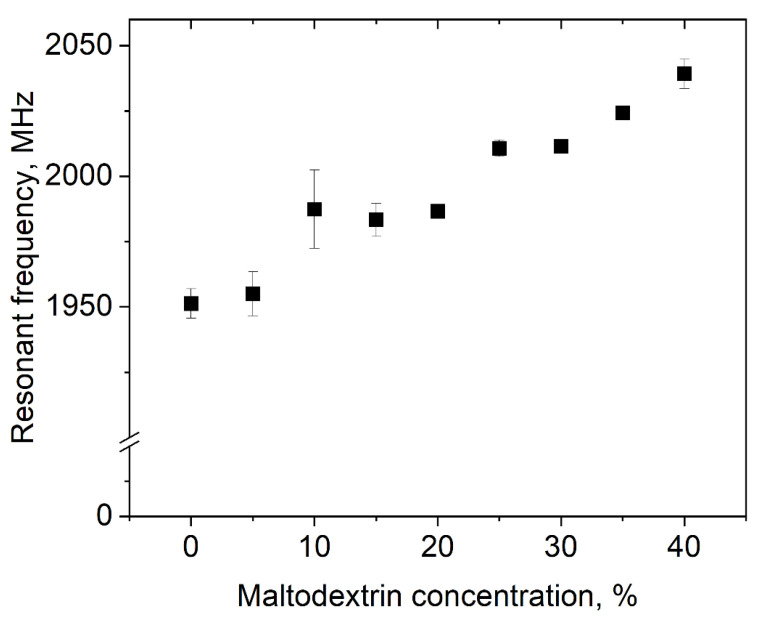
Impact of MD concentration on the resonant frequency of dispersions with 3% polysorbate 80.

In Figure 6, the resonant frequency of polysorbate 80 dispersions with an MD content between 0 and 40% is shown. It was observed that with increasing sugar content, the resonant frequency shifted from 1951.3 ± 5.7 to 2039.2 ± 5.6 MHz. This seems to be plausible, as the addition of organic molecules increases the resonant frequency of water [33].

Summing up the results on the dielectric properties of the dispersion, the samples were able to convert radiation more efficiently into heat at higher sugar contents. The faster and concentrated heating resulted in harsher heating conditions. Nevertheless, we observed better preservation of shape and structure at higher MD contents. Hence, the dielectric properties of the complex dispersion do not explain the collapse of the foam during the MWVD process.

**Figure 7:**
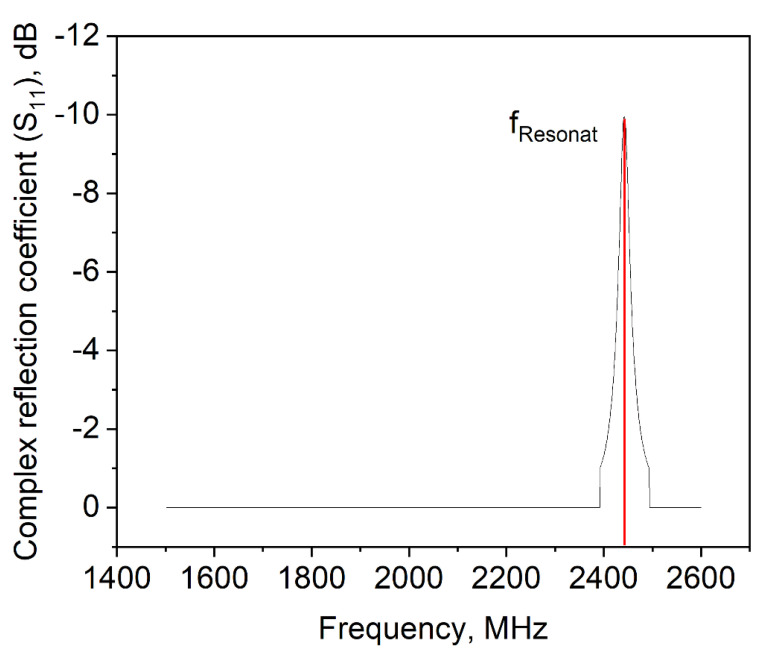
Complex reflection coefficient *S_11_* of pure polysorbate 80.

**Figure 8:**
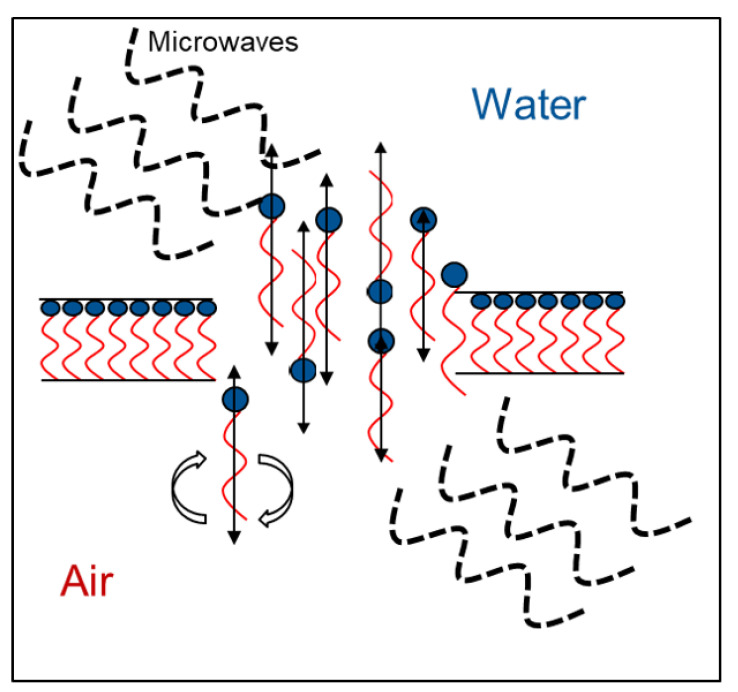
Schematic representation of the behavior of polysorbate 80 at the air/water interface during foam drying.

In order to more deeply address this question, different concentrations of MD without a foaming agent (i.e., polysorbate 80) were tested (data not shown), but they did not give a hint for the drying behavior of the samples. Hence, a dispersion of 100% foaming agent was examined in view of its dielectric properties. In Figure 7, the complex reflection coefficient *S_11_* depending on the frequency is shown. This value represents the interactions of a material with the electromagnetic wave if it is impinged with a certain frequency. The frequency where the interactions are the strongest is called resonant frequency, which is at 2445 MHz for the pure surfactant. This matches the frequency band which the microwave drying plant emits to apply power to the sample (i.e., 2450 MHz ± 50 MHz). Therefore, it appears that a so-called frequency catastrophe happened at the air/water interface, as schematically shown in Figure 8. Due to the overlapping microwave and resonant frequencies, the ethylene group of the surfactant is polarized very efficiently and at a high frequency. Further, interactions between water and the ethylene groups of polysorbate might be affected by the radiation [34], which might result in lower interface stability. Due to the high applied power, the movement of the surfactant caused hole formation within the surfactant layer. This in turn resulted in a collapse of the foam structure during microwave application. 

For high sugar concentrations and, consequently, thick lamellae, this effect can be suppressed by the high viscosity of the bulk phase. However, due to the quick expansion of the air bubbles during the vacuum and heating process, it could be that the counteracting forces become too weak. In a first step, the air bubbles would show coalescence with other bubbles until the necessary amount of surfactant to stabilize the interface is too low. Then, the bubble and the foam would collapse. 

Observations during the drying process showed that the foam started to collapse when the microwave power was on and stopped decaying right after turning the microwave power off (Figure 1). This indicates strongly that the resonant frequency of the foaming agent had a strong impact on the foam decay and not the overall dielectric properties of the bulk phase. Finally, the dielectric properties of polysorbate 80 seem to be one of the main reasons for the foam decay during MWVD.

## 4. Conclusions

In this study, the behavior of polysorbate 80–maltodextrin foams during vacuum drying processes with different heating methods was investigated. It was shown that there is not one single parameter that is overall important to achieve a successful drying process. Nevertheless, surface dilatational rheology seems an important parameter because a certain elasticity (tan(*φ*) < 0.4) of the surface is mandatory to withstand the mechanic stress during the evacuation of the drying plant. For the case of MWVD, it was not clear if just the dielectric properties of the surfactant had this massive impact on foam stability during the drying process. However, the results indicate that the dielectric properties of the foaming agent and the surface dilatational rheology of the complex system are responsible for its behavior during microwave-assisted processes, which is a new insight related to microwave-assisted drying. Further, a certain ratio of *E”/E’* expressed as tan(*φ*) < 0.2 was shown to be necessary for successful drying with MWVD, and the resonant frequency of the foaming agent should not match the working frequency of the microwave drying plant (2450 MHz ± 50 MHz). As the dielectric properties change with the temperature, it would be interesting to investigate these parameters at different, commonly used temperatures for vacuum drying. Further, it would be of interest to determine the importance of effects at the molecular level to better understand the behavior of foam during the drying process. Besides surfactants, proteins are commonly used as foaming agents, which is why we investigated the behavior of protein samples during MWVD in a second study. Further, it would be of interest to use other microwave sources to determine the specific effect of the microwave frequency.

## Figures and Tables

**Table 1:** Calculated tan(*φ*) of samples with 3% polysorbate 80 and an MD content between 0 and 40% at 20 °C and an amplitude of 500‰.

c(MD), %	0	5	10	15	20	25	30	35	40
tan(*φ*), -	0.20	0.24	0.45	0.46	0.53	0.50	0.48	0.40	0.28

**Table 2:** Calculated tan(*δ*) for dispersions with 3% polysorbate 80 and an MD content between 0 and 40% at 20 °C.

c(MD), %	0	5	10	15	20	25	30	35	40
tan(*δ*), -	0.12	0.13	0.14	0.16	0.17	0.20	0.22	0.23	0.26

## Data Availability

Data will be available upon request from the corresponding author.
